# Short-Term Heat Shock Affects Host–Virus Interaction in Mice Infected with Highly Pathogenic Avian Influenza Virus H5N1

**DOI:** 10.3389/fmicb.2016.00924

**Published:** 2016-06-15

**Authors:** Jia Xue, Xiaoxu Fan, Jing Yu, Shouping Zhang, Jin Xiao, Yanxin Hu, Ming Wang

**Affiliations:** ^1^Key Laboratory of Animal Epidemiology and Zoonosis of Ministry of Agriculture, College of Veterinary Medicine, China Agricultural UniversityBeijing, China; ^2^China Animal Health and Epidemiology CenterQingdao, China; ^3^Tianjin Entry-Exit Inspection and Quarantine BureauTianjing, China; ^4^Department of Immunology, College of Animal Science and Veterinary Medicine, Henan Institute of Science and TechnologyXinxiang, China; ^5^Key Laboratory of Veterinary Bioproduction and Chemical Medicine of the Ministry of Agriculture, Zhongmu Institutes of China Animal Husbandry Industry Co. Ltd.Beijing, China

**Keywords:** influenza A virus, short-term heat short, heat shock response, HSP70, cytokine

## Abstract

Highly pathogenic avian influenza virus (HPAIV) H5N1 is a highly contagious virus that can cause acute respiratory infections and high human fatality ratio due to excessive inflammatory response. Short-term heat shock, as a stressful condition, could induce the expression of heat shock proteins that function as molecular chaperones to protect cells against multiple stresses. However, the protective effect of short-term heat shock in influenza infection is far from being understood. In this study, mice were treated at 39°C for 4 h before being infected with HPAIV H5N1. Interestingly, short-term heat shock significantly increased the levels of HSP70 and pro-inflammatory cytokines IL-6, TNF-α, IFN-β, and IFN-γ in the lung tissues of mice. Following HPAIV H5N1 infection, short-term heat shock alleviated immunopathology and viral replication in lung tissue and repressed the weight loss and increased the survival rate of H5N1-infected mice. Our data reported that short-term heat shock provided beneficial anti-HPAIV H5N1 properties in mice model, which offers an alternative strategy for non-drug prevention for influenza infection.

## Introduction

The global prevalence of highly pathogenic avian influenza virus (HPAIV), with startling rates of avian morbidity and mortality, has a major socioeconomic impact in recent decades. Particularly, the H5N1 subtype of HPAIV causes pneumonia leading to acute respiratory distress syndrome and even death in humans (Peiris et al., 2007). The transmission and infection of HPAIV H5N1 from infected avian sources have been a potential threat to public health (Bi et al., 2015). Recent evidence showed that the host immune response to influenza virus infection involved the production of inflammatory cytokines, which play a critical role in both innate and adaptive immunity (Sladkova and Kostolansky, 2006; McGill et al., 2009; Kreijtz et al., 2011; Iwasaki and Pillai, 2014; Killip et al., 2015). Cytokines exert antiviral effects, but an overactive inflammatory response might contribute to immunopathology (Peiris et al., 2009). Patients infected by HPAIV H5N1 have higher serum levels of pro-inflammatory cytokines (e.g., IL-6, TNF-α) compared to those with seasonal influenza. Correspondingly, higher death rate has been reported in HPAIV-infected people with elevated amounts of these cytokines than patients with less hyper secretion (de Jong et al., 2006). Similarly, mice have shown increasing levels of various cytokines including IL-6, IFN-γ and TNF-α after being infected by H5N1 HPAIV (Szretter et al., 2007). The underlying mechanism of “cytokine storm” is associated with recruitment of neutrophils and macrophages into the lungs, which leads to acute lung inflammation (Perrone et al., 2008). Therefore, the current strategy for prevention and treatment of influenza infection focuses on the alleviation of the pathological injuries due to overreaction of immune system caused by the cytokine storm (Dunning et al., 2014).

Heat shock, exposure to oxidative stress, nutritional deficiencies, ultraviolet irradiation, chemicals, viral infections, etc., stimulate the expression of heat shock proteins (HSPs; Lindquist, 1986). It has been demonstrated that appropriate heat shock confers robust host immunity against the infection of certain bacteria (Wojda and Taszlow, 2013; Taszlow and Wojda, 2015) or viruses (Buccellato et al., 2007; Cevallos and Sarnow, 2010; Honda et al., 2015; Merkling et al., 2015). In the study of *Galleria mellonella* larvae model, host treated with short-term heat shock before *Bacillus thuringiensis* infection modulated the immune response and presented a preferentially outcome of recovery compared to the non-shocked, infected counterparts (Wojda and Taszlow, 2013). The response to heat shock is mechanically characterized by the production of HSPs, particularly HSP70. HSPs serve as molecular chaperones to protect cells against a multitude of stresses. Moreover, HSPs induced by one type of stimulus may initiate protection against other types (Lanneau et al., 2010). HSPs play an essential role in both innate and adaptive immunity; among which, this protein family activates monocytes, dendritic cells, and macrophages, induces the production of pro-inflammatory cytokines (IL-6, TNF-α) secretion, and favors the process of antigen presentation for cell-mediated immune response (Asea et al., 2000, 2002; Murshid et al., 2012). Accumulating studies suggest that HSP70 can bind with RNP complex and block influenza A virus replication *in vitro* and *in vivo* (Hirayama et al., 2004; Li et al., 2011). However, whether short-term heat shock, which induces high-level expression of HSP70, could protect against influenza virus warrants further investigation. In this study, we examined the protective role of short-term heat shock in HPAIV H5N1-infected mice. Our data showed that short-term heat shock ameliorated the pulmonary immunopathology and increased the survival rate of H5N1-infected mice.

## Materials and methods

### Mice

Female BALB/c mice, 6–8 weeks old, were purchased from Vital River Laboratories (Beijing, China), and the original breeding pairs were purchased from Charles River Laboratories (Beijing, China). The mice were raised in independent ventilated cages and received pathogen-free food and water. Animal experiments were performed according to the Regulations of Experimental Animals of Beijing Authority and approved by the Animal Ethics Committee of the China Agriculture University.

### Virus and challenge

The H5N1 influenza virus (A/Chicken/Henan/1/2004) used in this study was isolated from infected chicken flocks. The virus was grown in the allantoic cavities of 10-day-old embryonated chicken eggs. The allantoic fluid was collected and centrifuged to remove debris before aliquoting and rapid freezing for storage at −80°C until use. The 50% lethal dose (LD_50_) was determined in mice before use. Virus stocks were diluted in phosphate-buffered saline (PBS). Mice were anesthetized with Zoletil (Virbac, Carros, France). For infection, virus was diluted to 3 LD_50_ in 50 μl of PBS and instilled intranasally into anesthetized mice. Mice were euthanized at various time points and the lung tissues were collected. All experiments with H5N1 virus were conducted in a Biosafety Level 3 (BSL-3) containment laboratory approved by the Ministry of Agriculture of China.

### Short-term heat shock

Mice were randomly divided into heat-shocked and non-shocked groups. Mice in the heat-shocked group were placed in a biological oxygen demand (BOD) incubator and subjected to short-term heat exposure for 1, 2, 4, 6, and 8 h at 39 ± 1°C with 35 ± 5% air humidity. Mice in the non-shocked group were kept at room temperature (25°C).

### SDS–PAGE and immunoblotting

Mice lung tissues were lysed in RIPA buffer (Beyotime, Haimen, Jiangsu, China) with 10 mM PMSF (Beyotime, Haimen, Jiangsu, China) and 20 mM Cocktail (Roche) on ice for 20 min. The lysates were then centrifuged at 4°C for 5 min at 10,000 *g* in a microcentrifuge to remove cell debris. Proteins were extracted from the supernatant. Total protein [30 μg; as determined by BCA protein assay (Applygen, Beijing, China)] was boiled for 5 min and resolved in 12% polyacrylamide gels and transferred to polyvinylidene fluoride (PVDF) membranes (Millipore, Bedford, MA, USA). The blotted membranes were incubated in 5% skim milk to block nonspecific absorption of antibodies. The membranes were then immunoblotted with the following antibodies and dilutions: anti-HSP 70 (SC-66048) 1:200, anti-β-actin (SC-1615) 1:5000, goat anti-mouse IgG-HRP (sc-2005) 1:2000, and donkey anti-goat IgG-HRP (SC-2020) 1:5000 (each Santa Cruz Biotechnology Inc., Santa Cruz, CA, USA). Proteins were detected using Western Lightning Chemiluminescence Reagent Plus (Perkin–Elmer Life Sciences, Inc., Norwalk, CT, USA) and visualized by exposure to Fuji medical X-ray film (RX-U; Fuji Photo Film Co., Tokyo, Japan).

### Plaque assay

MDCK cells were cultured in Dulbecco's modified Eagle's medium (DMEM; Hyclone Laboratories, Beijing, China) containing 10% fetal bovine serum (Hyclone Laboratories, Beijing, China), 100 U/ml penicillin, and 100 μg/ml streptomycin. The supernatants of mice lung tissues were serially diluted in 10-fold steps and added to a monolayer of MDCK cells in semi-solid agar that contained 0.5 μg/ml TPCK-trypsin (Sigma-Aldrich, St Louis, MO, USA) and DMEM. MDCK cells were incubated at 37°C in 5% CO_2_ for 72 h and then fixed and stained with 1% crystal violet for plaque count.

### Histopathological analysis

Tissues were harvested and fixed with 4% neutral formalin at room temperature for 48 h. The serial tissue sections were cut to 5 μm thickness after embedding in paraffin. Slides were stained with hematoxylin and eosin (H&E) and examined by light microscopy (BX41; Olympus, Tokyo, Japan).

### RT-qPCR

Total RNA was prepared from mice lung tissues homogenized in TRIzol (Invitrogen, Carlsbad, CA, USA) according to the manufacturer's instructions. RNA (0.2 μg) was reverse transcribed into cDNA.

Expression of the hemagglutinin (HA) gene of H5N1 influenza virus was quantified using a Power SYBR^®^ Green PCR Master Mix Kit (Applied Biosystems, Foster City, CA, USA). The following primers were used for qPCR of HA: forward primer, 5′-CGCAGTATTCAGAAGAAGCAAGAC-3′; and reverse primer, 5′-TCCATAAGGATAGACCAGCTACCA-3′. Prepare the reaction as follow: cDNA 5 μl, Master Mix 10 μl, forward/reverse primer (25 μM) 0.5 μl each, and dH_2_O 4 μl to 20 μl total. The reaction was run on an ABI 7500 thermal cycler with an initial denaturation step at 95°C for 10 min, followed by 40 cycles of 95°C for 15 s, 56°C for 30 s, and 72°C for 45 s. Data analysis was performed using the 7500 v. 2.0 software (Applied Biosystems, Foster City, CA, USA). The HA gene copy number was calculated using a HA-containing plasmid of known concentration as a standard.

The expression levels of the following genes were also quantified by qPCR:

β-actin F: 5′-GAGACCTTCAACACCCCGC-3′
β-actin R: 5′-ATGTCACGCACGATTTCCC-3′
IL-6 F: 5′-AGCCAGAGTCCTTCA-3′
IL-6 R: 5′-TCTTGGTCCTTAGCC-3′
TNF-α F: 5′-GTAGCCCACGTCGTAGCAAA-3′
TNF-α R: 5′-CCCTTCTCCAGCTGGAAGAC-3′
IFN-β F: 5′-TCCAGCTCCAAGAAAGGACG-3′
IFN-β R: 5′-GCATCTTCTCCGTCATCTCC-3′
IFN-γ F: 5′-AGTGGCATAGATGTGGAA-3′
IFN-γ R: 5′-GACCTGTGGGTTGTTGA-3′
HSP70 F: 5′-TGAGCAGCCCATCCTTAGTG-3′
HSP70 R: 5′-ATAGGCATCCGTCCCTTTGT-3′

Prepare the reaction as follow: cDNA 2 μl, Master Mix 10 μl, forward/reverse primer (25 μM) 0.5 μl each, and dH_2_O 7 μl to 20 μl total. Reactions were carried out on an ABI 7500 instrument with initial denaturation at 95°C for 10 min, then 40 cycles of denaturation at 95°C for 15 s, annealing at 56°C for 30 s, and extension at 72°C for 45 s. Gene expression was normalized using the 2^−Δ*ΔCT*^ method with β-actin as an internal standard.

### ELISA

The IL-6, TNF-α, IFN-β, and IFN-γ concentrations in homogenized lung tissues were determined using ELISA kits (eBioscience, San Diego, CA) according to the manufacturer's instructions.

### Statistical analysis

Statistical analyses were performed as indicated in the figures. All data were included in the analyses. GraphPad Prism version 6.00 (San Diego, CA, USA, www.graphpad.com) was used for analysis.

## Results

### Short-term heat shock is associated with increased survival and reduced weight loss in HPAIV-infected mice

**Figure 1:**
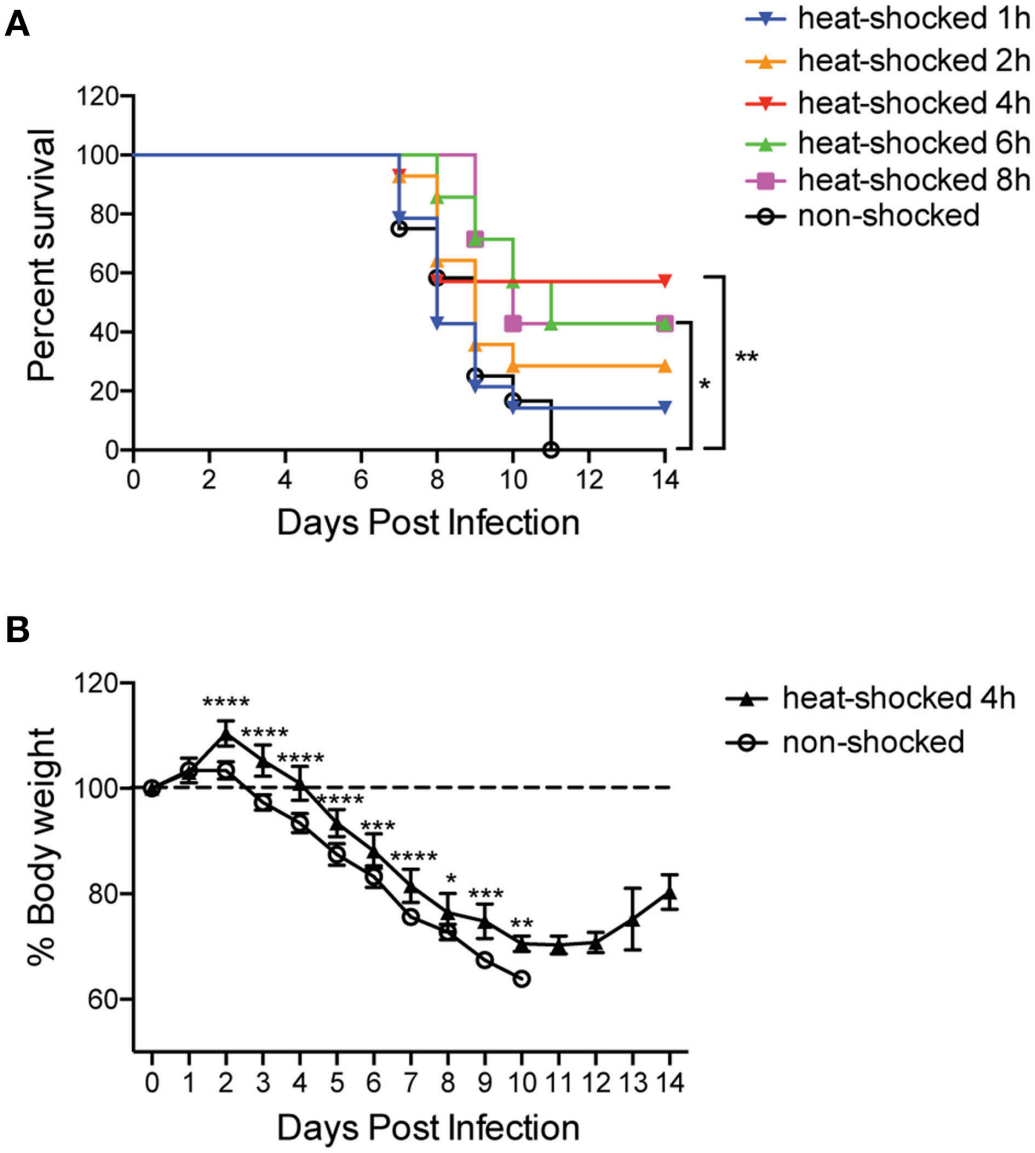
**Short-term heat shock increased the survival rate and reduced weight loss in HPAIV-infected mice**. Mice were randomly divided into heat-shocked and non-shocked groups. Mice in the heat-shocked group were exposed to 39°C for 1, 2, 4, 6, and 8 h before being infected with 3 LD_50_ H5N1 HPAIV intranasally. Mice in the non-shocked group were maintained at room temperature (25°C). After the treatment, all the mice were maintained at room temperature. **(A)** The survival rate of each group was monitored for 14 days post-infection (*n* = 12–14 per group total; *p < 0.05, **p < 0.01 by log-rank test). **(B)** Mice weight loss was monitored overtime (*n* = 7 per group; *p < 0.05, **p < 0.01, ***p < 0.001, ****p < 0.0001 by two-way ANOVA with Sidak's multiple comparisons test).

To determine the effect of short-term heat shock on HPAIV H5N1 infection, mice were exposed to 39°C before or after being inoculated intranasally with 3 LD_50_ virus. As shown in Figure 1A, the mice from non-shocked group began to die at Day 7 and the mortality rate successively increased till Day 11. In contrast, the treatment of heat shock for 1, 2, 4, 6, and 8 h before the infection exerted protection toward mice, with the survival rates of 17, 29, 57, 43, and 43% at 11 dpi, respectively. The result also suggested that heat shock for 4 h prior to the infection might play a beneficial role in the protection for mice against lethal influenza infection. On the basis of this finding, we identified the loss of body weight during the infection and found a tendency of milder loss of weight in the mice pretreated with heat shock for 4 h than that in mice without treatment from 1 to 10 days post-infection. Notably, the weight of heat-shocked mice began to gain weight at Day 12 post-infection, but the non-treated mice died by Day 11 post-infection (Figure 1B). In this scenario, our data indicated that a 4-h treatment of heat shock prior to infection contributed to an alleviating effect on H5NI-infected mice.

### Short-term heat shock reduced the replication of HPAIV H5N1

**Figure 2:**
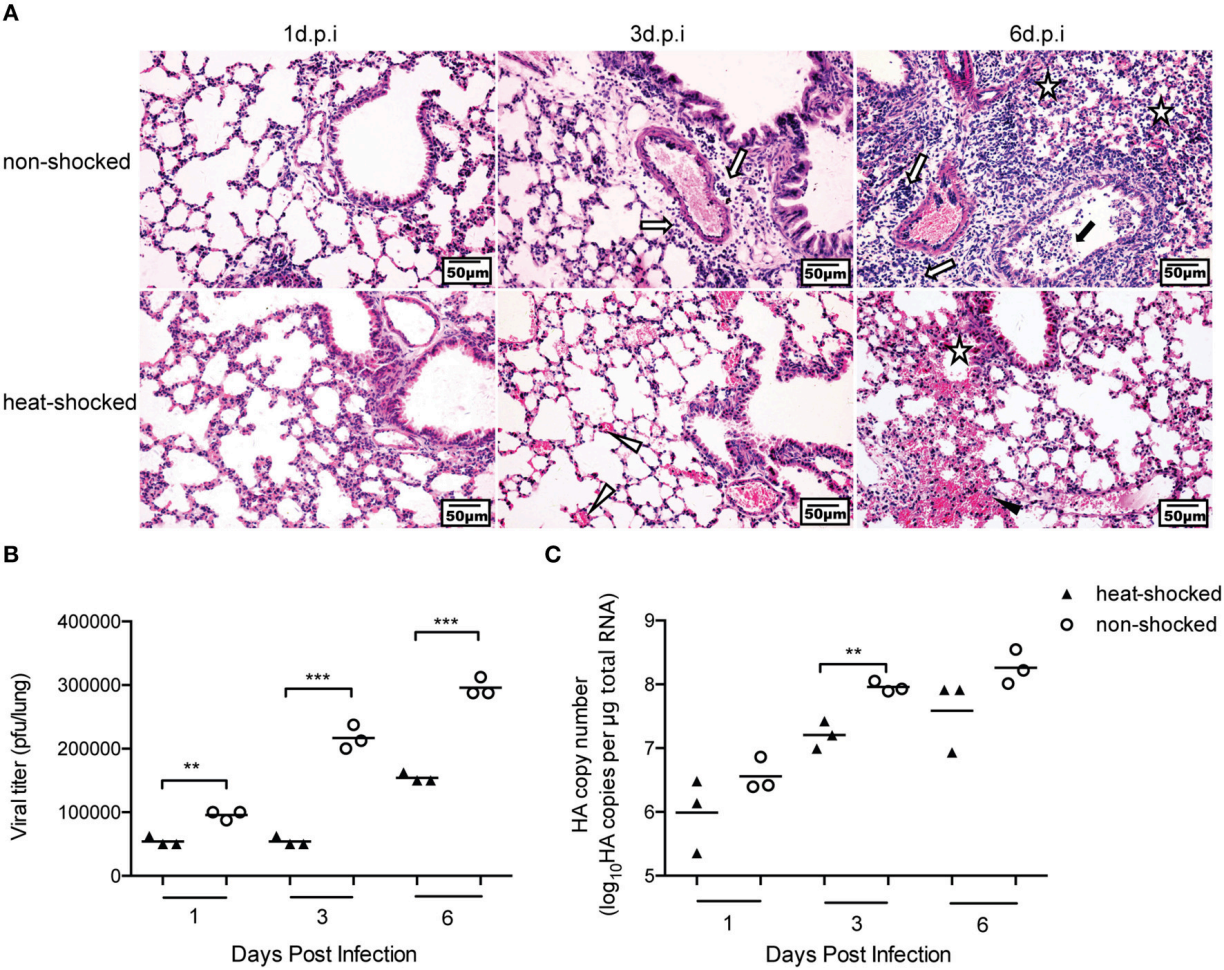
**Short-term heat shock decreased the virulence of H5N1 HPAIV in infected mice**. Mice in the heat-shocked group were exposed to 39°C for 4 h before being infected with 3 LD_50_ H5N1 HPAIV intranasally. Mice were sacrificed at Days 1, 3, and 6 post-infection for harvesting of lung tissues. Representative sections from each group were stained by H&E (Bar = 50 μm). The open arrow indicates interstitial edema and inflammatory cell infiltration around small blood vessels; the solid arrow indicates dropout of mucous epithelium in the bronchioles; the open triangle indicates pulmonary congestion; the solid triangle indicates infiltration of the alveolar lumen by erythrocytes; the star indicates infiltration of the alveolar lumen by inflammatory cells **(A)**. Three mice in each group were euthanized for measurement of viral titer in the lungs by plaque assay **(B)**, and real-time PCR **(C)** (**p < 0.01, ***p < 0.001 by two-way ANOVA with Sidak's multiple comparisons test).

Following the challenge with HPAIV, histological changes in the lungs of heat-shocked and non-shocked mice at Days 1, 3, and 6 post-infection were evaluated. On Day 1 post-infection, no apparent histological changes in lungs from both groups were observed. On Day 3 post-infection, however, the lungs of mice in the non-shocked group were characterized by interstitial edema and inflammatory cell infiltration around small blood vessels, and exfoliation of the bronchiolar epithelium. In comparison, the heat-shocked mice showed mild lung lesions characterized by only pulmonary congestion. Progressively, on Day 6 post-infection, the lung lesions in non-shocked mice showed extremely marked histological changes, with detachment of the bronchiolar mucous epithelium, thickening of alveolar walls, as well as flooding of alveolar lumen with edema fluid mixed with exfoliated alveolar epithelial cells, erythrocytes, and inflammatory cells. Nevertheless, the heat-shocked mice only presented mild lung lesions characterized by infiltration of the alveolar lumen by a few erythrocytes and inflammatory cells (Figure 2A).

As susceptibility to H5N1 virus infection is correlated with the increase of viral titer, we also assessed viral replication in heat-shocked and non-shocked mice using plaque assays and real-time PCR analysis of the viral HA gene. Mice were sacrificed at Days 1, 3, and 6 post-infection, and the viral loads in the lungs were determined. Compared with the non-shocked group, statistically fewer infectious viral particles were detected by plaque assay in the lungs of mice in the heat-shocked group (Figure 2B). Consistent with the plaque assay data, the results of qPCR unraveled a reduced accumulation of HA gene copies on premises with heat shock (Figure 2C). These results suggest that short-term heat shock could relieve the influenza-induced lung damages by limiting viral replication.

### Short-term heat shock increased the expression of heat shock protein 70 and inflammatory cytokines in mice

**Figure 3:**
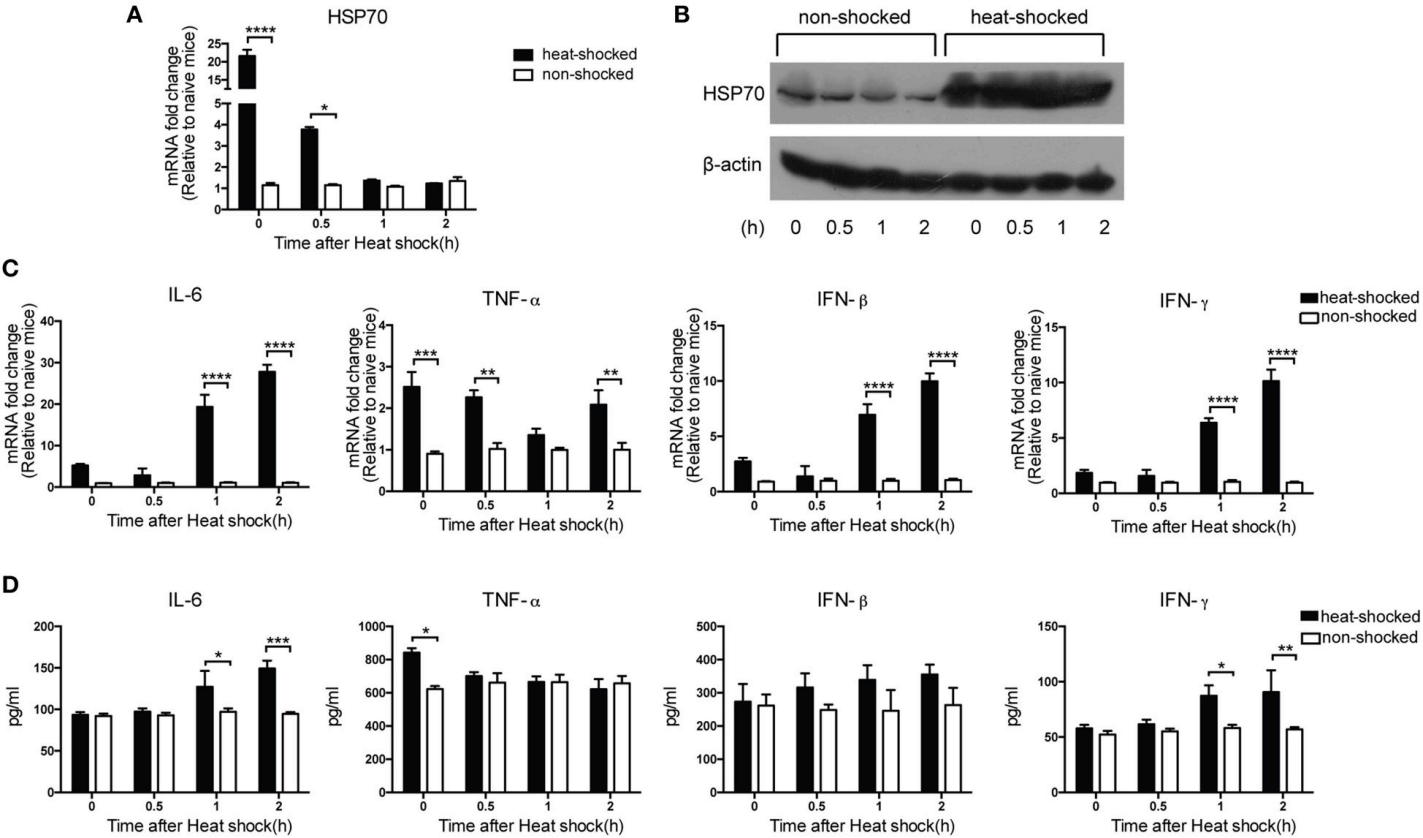
**Short-term heat shock increased the expression of HSP70 and inflammatory cytokines in mice**. Mice were exposed to 39°C for 4 h and then transferred to room temperature and sacrificed at 0, 0.5, 1, and 2 h after heat shock treatment for lung tissues collection. The non-shocked group was maintained at room temperature. HSP70 expressions were measured by qPCR **(A)** and western blot **(B)**. IL-6, TNF-α, IFN-β, and IFN-γ expressions were determined by qPCR **(C)** and ELISA **(D)**. Gene expression was normalized with β-actin as an internal standard and then compared with the naïve mice (*n* = 3–5, *p < 0.05, **p < 0.01, ***p < 0.001, ****p < 0.0001 by two-way ANOVA with Sidak's multiple comparisons test).

To determine the expression of HSP70 of mice in response to short-term heat shock, we collected mice lung tissues of each time point after heat shock. We found that the expression of HSP70 mRNA in heat-shocked mice was immediately elevated after the treatment, which was significantly different compared to that in non-shocked mice (Figure 3A). We kept measuring the mRNA level for HSP70 at 4, 6, and 8 h till 12 h after heat shock. However, there were no significant differences between the two groups (data not shown). The expression of HSP70 protein in the lung tissues of the heat-shocked mice was also determined by western blot. The result showed that after heat shock for 4 h, the levels of HSP70 protein in lungs were increased and maintained for 2 h at room temperature (Figure 3B). We also evaluated the innate immune response by detecting cytokine levels in mice lung tissues after the treatment. Compared with the non-shocked mice, 4-h heat shock treatment increased the mRNA level of TNF-α immediately. However, the increment of the mRNA levels of IL-6, IFN-β, and IFN-γ in the lung tissues could be detected at 1 and 2 h after the heat shock treatment (Figure 3C). Furthermore, the ELISA results of pro-inflammatory cytokines were in agreement with the difference of mRNA levels (Figure 3D), implying that short-term heat shock induced the expressions of HSP70 and pro-inflammatory cytokines and interferons, thereby promoting immediate defense of the innate immune response.

### Modulation of heat shock on the expression of heat shock protein 70 and inflammatory cytokines in HPAIV-infected mice

**Figure 4:**
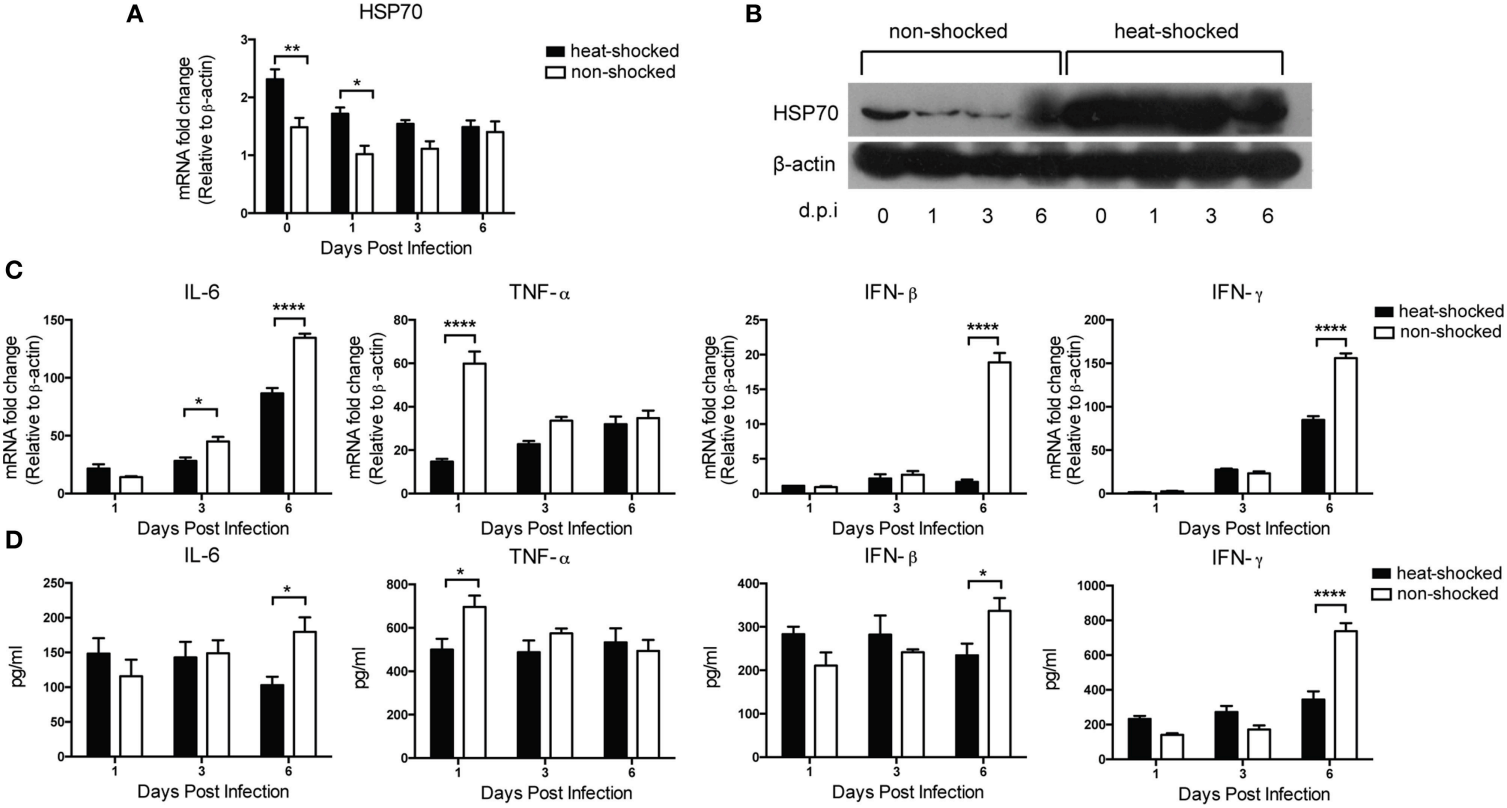
**Effect of heat shock treatment on HSP70 and inflammatory cytokines expression in H5N1-infected mice**. Mice in the heat-shocked group were exposed to 39°C for 4 h before being infected with 3 LD_50_ H5N1 HPAIV intranasally. Mice in the non-shocked group were maintained at room temperature (25°C). After infection, all the mice were maintained at room temperature. Mice were sacrificed at Days 0, 1, 3, and 6 post-infection for lung tissues harvest. HSP70 expression was determined by qPCR **(A)** and western blot **(B)**. IL-6, TNF-α, IFN-β, and IFN-γ expressions were determined by qPCR **(C)** and ELISA **(D)**. Gene expression was normalized with β-actin as an internal standard (*n* = 3–5, *p < 0.05, **p < 0.01, ****p < 0.0001 by two-way ANOVA with Sidak's multiple comparisons test).

In order to investigate the effect of short-term heat shock on HSP70 expression in H5N1-infected mice, we detected the expression of HSP70 as well as cytokines including IL-6, TNF-α, IFN-β, and IFN-γ with real-time PCR, western blot, and ELISA at Days 0, 1, 3, and 6 post-infection. The expression of HSP70 in heat-shocked mice was significantly induced at 0 and 1 dpi compared to that in non-treated controls. While a decreasing trend of level was observed, the levels in the heat-shocked group were still higher than that in the non-shocked group at 3 and 6 dpi, indicating that HSP70 possesses protective function especially for early-onset infection (Figures 4A,B). Following the H5N1 infection, mice with heat-shocked treatment showed markedly lower level of TNF-α mRNA at Day 1 than the mice without the treatment (Figure 4C). Intriguingly, relatively impaired expressions of IL-6, IFN-β, and IFN-γ were observed in treated mice at Day 6 compared to that in the non-shocked mice (Figures 4C,D). The HSP70 result along with pro-inflammatory cytokines analysis demonstrated that short-term heat shock positively modulated the expression of HSP70 and downregulated the overexpression of inflammatory cytokines during the lethal infection of HPAIV, which could alleviate lung immune injury in HPAIV-infected mice.

## Discussion

In previous study, we reported that chronic heat shock significantly suppressed innate immunity, resulting in the growing rate of mortality and viral loads in the lungs of mice infected with HPAIV H5N1 (Jin et al., 2011). However, in the present study, the exposition of mice to short-term heat shock resulted in the increment of IL-6, TNF-α, IFN-β, and IFN-γ, and their resistant ability to HPAIV H5N1 infection. It has been demonstrated that IL-6 plays a vital role as innate immune cytokine in providing protection against influenza A infection (Dienz et al., 2012) and TNF-α has antiviral activity in lung epithelial cells (Seo and Webster, 2002). It is also known that both type I and type III IFNs contribute to protection against influenza virus infection (McNab et al., 2015). Moreover, pre-immune state induced by IFN-γ could generate an antiviral response against influenza virus (Yuk et al., 2016). However, the antiviral activity of the cytokines is a two-sided coin. The overexpression of pro-inflammatory cytokines including interferons, TNFs, and interleukins has been associated with pathogenesis during influenza virus infection in humans and animal models (Szretter et al., 2007; Peiris et al., 2009; Darwish et al., 2011). Cytokine storm associated with excessive release of pro-inflammatory cytokines leads to an acute mononuclear/neutrophilic inflammatory response followed by a chronic fibro proliferative phase in the lung (Tisoncik et al., 2012). In this study, we noticed that the immunopathological injuries in lung tissues in short-term heat-shocked mice were strikingly alleviated after infection. The alleviated lung injury of HPAIV-infected mice was manifested by less replication of the virus and the restricted expression of IL-6, TNF-α, IFN-β, and IFN-γ.

Cumulative studies have shown that HSP70 plays an essential protective role during lung inflammation and immune injury, via the activation of both the innate and adaptive immunity either directly or indirectly (Jacquier-Sarlin et al., 1994; Wallin et al., 2002; Wheeler and Wong, 2007). Recent progresses provided further knowledge about HSP70 against influenza infection and demonstrated that it interacted with PB2 and PB1 proteins and translocated into the nuclei of A549 cells upon infection for blocking the ongoing replication of virus (Li et al., 2011). Consistent with the previous study that the expression of HSP70 can be induced by prostaglandin A1 or heat shock treatment (Hirayama et al., 2004), our data showed that short-term heat shock upregulated HSP70 levels in lung tissues of mice, which provides benefits in the defense against the ensuing flu attack. These findings highlight the potential role of heat shock-induced HSP70 in the antiviral response of the host.

Organisms must survive in the presence of a variety of stressors (Macario and Conway de Macario, 2005; Richter et al., 2010), though some of the stressors can lead to death of the organisms, depending on their duration and severity (Lindquist, 1986). Based on this information, fomentation, sauna, and infrared therapies, as sorts of physical stimuli, have been traditionally used to treat a variety of physical disorders in diverse cultures for thousands of years. Especially under the imbalanced condition within the body, people prefer to take fomentation, sauna, or infrared therapies to restore the physical functions (Anderson and Srivastava, 2000). A common mode of fomentation, sauna, and infrared therapies incorporates short-term heat shock of different levels and sources of heat. Nevertheless, the mechanism by which this may occur remains to be investigated. Previous studies suggested that host exposed to short-term heat shock directly before bacterial infection was triggered a potent immune response (Wojda and Taszlow, 2013; Taszlow and Wojda, 2015). In a similar fashion, our data demonstrated that short-term heat shock protected mice from H5N1 influenza virus infection, suggesting that heat stress may be feasible for the prevention of pathogenic microorganisms-associated disease.

Taken together, we demonstrated that short-term heat shock presented beneficial anti-HPAIV H5N1 properties in mice model. To our knowledge, this is the first report on the role of short-term heat shock prior to the influenza infection, which initiates host prompt antiviral response. Our finding shows potential for the rational development of alternative non-drug prevention strategies for influenza infection. Further functional experiments with adequate *in vivo* knockout models are needed to explore the role of HSP70 and relative cytokines induced by heat shock in the suppression of viral replication.

## Author contributions

JX performed experiments and wrote the manuscript, XF, JY, JX, SZ performed experiments, YH and MW wrote the article and conceived the experiments.

### Conflict of interest statement

The authors declare that the research was conducted in the absence of any commercial or financial relationships that could be construed as a potential conflict of interest.
